# Development of a New Detection Algorithm to Identify Acute Coronary Syndrome Using Electrochemical Biosensors for Real-World Long-Term Monitoring

**DOI:** 10.3390/bioengineering8020028

**Published:** 2021-02-20

**Authors:** Pau Redon, Atif Shahzad, Talha Iqbal, William Wijns

**Affiliations:** 1 CÚRAM: SFI Research Centre for Medical Devices, H91 W2TY Galway, Ireland; william.wijns@nuigalway.ie; 2 Smart Sensors Lab, School of Medicine, National University of Ireland, Galway (NUIG), H91 TK33 Galway, Ireland; atif.shahzad@nuigalway.ie (A.S.); talha.iqbal@nuigalway.ie (T.I.); 3 Centre for Systems Modelling and Quantitative Biomedicine, Institute for Metabolism and Systems Research, University of Birmingham, Edgbaston, Birmingham B15 2TT, UK; 4 Saolta University Healthcare Group, University Hospital Galway, Newcastle Road, H91 YR71 Galway, Ireland

**Keywords:** cardiac biomarkers, risk stratification algorithm, wearable device, electrochemical sensor, long-time monitoring

## Abstract

Electrochemically based technologies are rapidly moving from the laboratory to bedside applications and wearable devices, like in the field of cardiovascular disease. Major efforts have focused on the biosensor component in contrast with those employed in creating more suitable detection algorithms for long-term real-world monitoring solutions. The calibration curve procedure presents major limitations in this context. The objective is to propose a new algorithm, compliant with current clinical guidelines, which can overcome these limitations and contribute to the development of trustworthy wearable or telemonitoring solutions for home-based care. A total of 123 samples of phosphate buffer solution were spiked with different concentrations of troponin, the gold standard method for the diagnosis of the acute coronary syndrome. These were classified as normal or abnormal according to established clinical cut-off values. Off-the-shelf screen-printed electrochemical sensors and cyclic voltammetry measurements (sweep between −1 and 1 V in a 5 mV step) was performed to characterize the changes on the surface of the biosensor and to measure the concentration of troponin in each sample. A logistic regression model was developed to accurately classify these samples as normal or abnormal. The model presents high predictive performance according to specificity (94%), sensitivity (92%), precision (92%), recall (92%), negative predictive value (94%) and F-score (92%). The area under the curve of the precision-recall curve is 97% and the positive and negative likelihood ratios are 16.38 and 0.082, respectively. Moreover, high discriminative power is observed from the discriminate odd ratio (201) and the Youden index (0.866) values. The promising performance of the proposed algorithm suggests its capability to overcome the limitations of the calibration curve procedure and therefore its suitability for the development of trustworthy home-based care solutions.

## 1. Introduction

Over the past few decades, cardiovascular diseases (CVD) are on a continuous rise accounting for 17.64 million deaths worldwide in the general population [1]. Only in Europe, CVD causes every year 3.9 million deaths and an overall estimated cost of €210 billion, 53% corresponding to health care expenditure [2]. The unstoppable trend of CVD and other chronic diseases (e.g., diabetes, hypertension and obesity) in conjunction with a rapidly ageing population in the Western world is expected to increase the prevalence and mortality of associated diseases, like acute coronary syndrome (ACS) (e.g., myocardial infarction, stable and unstable angina). 

Such increase is expected to negatively impact the budget of the national health care systems not only due to an increase in the demand for biomarker testing (e.g., troponin) —only in the USA the estimated costs to diagnose ACS at Emergency Department (ED) ranges between $10–13 billion annually [3], but also worse patient outcomes due to the overflow of the ED, more adverse baseline conditions due to comorbidities or by longer turnover times resulting from an increase in the workload of specialized clinical laboratories. For every 30 min-delay to receive intervention the probability for 1-year mortality of acute myocardial infarction (AMI) survivors increases by 7.4% [4]. 

A potential solution to mitigate such impact could be the clinical implementation of telemonitoring strategies capable of precisely measuring levels of key cardiac biomarkers [5]. In the recent past, there has been a growing interest in developing minimally invasive wearable devices based on different biosensors (e.g., optical, electrochemical and magnetic) [6,7,8]. Out of all of them, the electrochemical biosensors have shown to be the most promising according to their miniaturization capabilities, low manufacturing costs and low power consumption [6]. According to Abdorahim et al. [7] “electrochemical immunosensing development is a recently developed and promising technology transitioning from the bench to the bedside”. Briefly, these can be classified as potentiometric, impedimetric, amperometric and conductimetric. While the first two respectively measure changes in voltage and impedance to determine the concentration of the analyte the two latter measure variations in current. 

Biosensors have continuously been improved to make them more sensitive to smaller variations of concentration, contributing to decrease the limit of detection (LOD) of the analytical assays. Even though lower LOD can contribute to faster diagnosis in low-risk patients it might have the opposite effect on high-risk patients, which are the ones demanding a more rapid and accurate diagnosis. Focusing on troponin detection-considered the gold standard method for detecting myocardial injury and tissue necrosis-major efforts have been made in developing new immobilization strategies and assay principle to detecting the I (cTnI) and the T (cTnT) isoforms of the troponin protein, see Table 1, in contrast with the limited efforts dedicated to developing new detection algorithms which are responsible for translating into concentration the changes in the measured signal or signals. Only three papers have focused on developing new detection algorithms [9,10,11,12]. 

Even though some recent studies have focused on the employment of machine learning algorithms to distinguish between analytes from cyclic voltammetry measurements [9,12], the most common method is the calibration curve. Generally speaking, this procedure translates the changes suffered by a measured signal into the concentration of the corresponding analyte through a univariate linear model. In other words, only one point of the corresponding measured signal is considered to determine the concentration of the analyte. Unfortunately, this can limit the operational range of an assay. Besides this limitation others are also worth considering when it comes to integrating these types of algorithms into wearable devices for long-term home-based monitoring, like:

1. Preservation of the biosensors;
2. Variability between batches of biosensors;
3. Effect of medications on the physical properties of blood;
4. Presence of interfering analytes in blood;
5. Sampling conditions affected by patient routine;
6. Variability between samples (e.g., different times of the day and sample volume).

All of them have consequences on the reliability of the measurements and can contribute to reducing the confidence of physicians and patients in these tools. Despite that some of these drawbacks can be mitigated when designing the device, with the associated cost increase, others are unpredictable or user-dependent. Consequently, there is a strong need to develop solutions that can contribute to generating trustworthy home-based care tools for their future implementation in clinical practice. 

In this context, the main objective is to develop a new detection algorithm oriented to electrochemical biosensors, for long-term and real-world monitoring, which can accurately stratify the risk of a patient suffering myocardial injury or tissue necrosis. Moreover, this detection algorithm should be aligned with good clinical practices and applicable to the general population as well as the high-risk population. 

## 2. Materials & Methods

**Figure 1:**
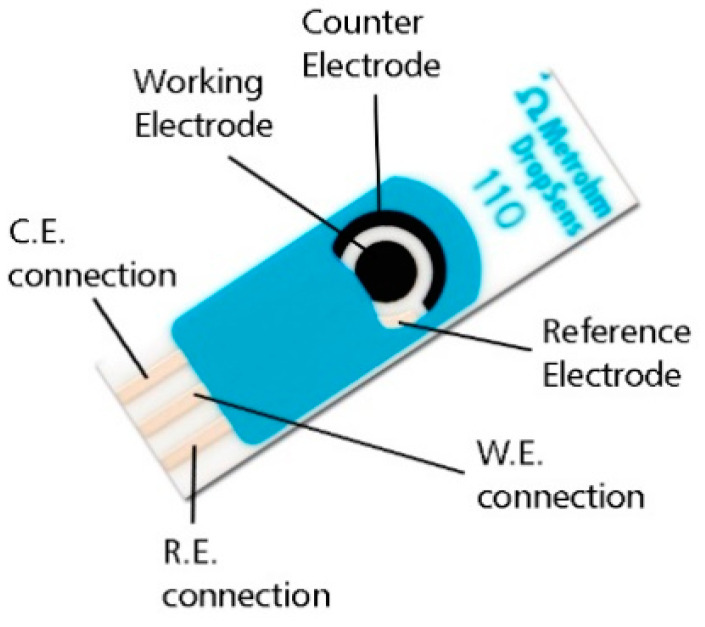
Schematic representation of the screen-printed electrodes used in this study.

One-hundred and twenty-three samples of phosphate buffer solution (PBS) were spiked with different concentrations of troponin, between 0 and 2000 pg/mL. Human cardiac troponin I-protein, antibody pair support kit and phosphate buffer sulfate (PBS) 1X Calcium and Magnesium free were purchased from Abbexa. The limit of detection (LOD) of the assay is 3000 pg/mL. The rationale for using cTnI instead of cTnT was because the former is cheaper, more accessible from a commercial point of view and most important of them all because it is released from less damaged cardiomyocytes [13] allowing to prevent or even mitigate unnecessary myocardial damage. Current clinical troponin cut-off values vary depending on the sensitivity of the assay [14,15,16,17]. In this manuscript, the samples were classified as normal or abnormal according to the threshold value commonly used for sensitive troponin assays, 0.03 μg/L [18]. A total of 45 samples (37%) had abnormal clinical values. The sensor employed was an off-the-shelf graphene screen-printed electrode, see Figure 1, which was functionalized with the corresponding antibodies following the recommendations of the supplier. The current-potential measurements at the surface of the sensor were performed using a Autolab PGSTAT 204 potentiostat/galvanostat device (MetroOhm, Runcorn, UK) [19]. These ranged from −1 to 1 V with a 5 mV step and were recorded in the following scenarios: (i) sensor + PBS, (ii) sensor + coating, (iii) sensor + coating + blocking and (iv) sensor + coating + blocking + troponin. These scenarios will be denoted from 1 to 4. To simulate potential variability between measurements all the processes were performed manually.

**Figure 2:**
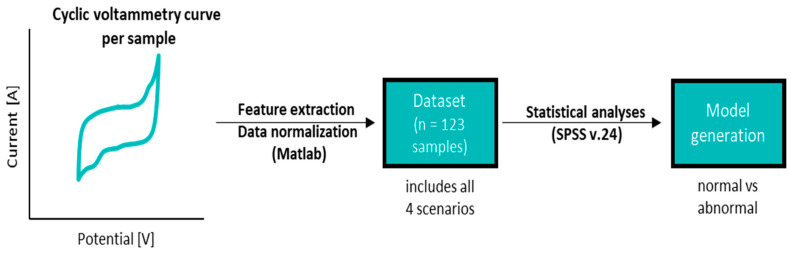
Description of the methodology followed in the manuscript.

The resulting potential-current curves for each sample were processed by the feature extraction algorithm developed by the authors using MATLAB software [20] and capable of identifying 60 different features. This process was followed by a normalization process of all variables. Statistical analyses (i.e., normality tests, Mann-Whitney U test and logistic regression model) were performed using IBM SPSS software v.24 (SPSS Software, Chicago, IL, USA) [21]. Figure 2 schematically illustrates the methodology used in this manuscript. The performance of the developed logistic regression model was evaluated making use of the confusion matrix and a broad number of key performance indicators (e.g., accuracy, specificity, recall or sensitivity, precision or positive predictive value (PPV), area under the curve (AUC), negative predictive value (NPV), F-Score, Youden index (YI), likelihood ratio positive (LR+), likelihood ratio negative (LR−) and diagnostic odds ratio (DOR)) [22,23,24]. 

The recall or sensitivity and precision or PPV are two very important parameters to assess the performance in identifying positive samples (i.e., in this case clinically abnormal samples). The former is the portion of real positive cases that are correctly predicted positive while the latter denotes the portion of predicted positive that is correctly real positive. On the other hand, the specificity and the negative predictive value (NPV) are respectively homologous of the two previously described parameters but assessing the performance of the model regarding its capability to identify the negative values or healthy subjects. Moreover, accuracy and F score are two frequently used parameters to assess overall performance. Accuracy is the portion of truly identified cases, independently of being positive or negative, from the total number of samples. The F score is the harmonic mean between PPV and sensitivity. Out of the three different types of averages that can be calculated (i.e., arithmetic, geometric and harmonic), the harmonic average is the most conservative of them all. The area under the curve (AUC) was also considered and calculated after performing a receiver-operator curve (ROC) analysis, in particular, the precision-recall plot. A higher AUC indicates that the model has a better capability to distinguish between clinically normal and abnormal samples. The likelihood ratio is a very valuable indicator to determine the accuracy of the diagnosis and is defined as the ratio of samples clinically abnormal and those considered as normal. LR+ is the best indicator for ruling-in diagnosis and vice-versa for LR−. The DOR is also a good indicator to measure the accuracy of the diagnosis. It is used to compare different diagnoses and is strongly dependent on the sensitivity and specificity of the model. Finally, YI is one of the oldest parameters to evaluate the diagnostic accuracy and can also be used to evaluate the discriminative power of several diagnosis methods. The closer it is to 1 the more discriminative it is.

## 3. Results

### 3.1. General Results

Initially, normality tests were performed on all the extracted features or variables. The results confirmed the lack of normal distribution. The following step was to identify the existence of significant differences between the readings of the different scenarios. To do so, the Mann-Whitney U test was performed to compare the readings between scenarios 1 and 4 and 3 and 4, see Table 2. Only the results of the variables included in the resulting logistic regression model (see Section 3.2 Logistic Regression Model) have been presented. The results of the remaining variables are available in Tables S1 and S2 of the Supplementary Files. The variables denoted as V1, V2 and V3 are greater for scenario 4 (V1: 52.75, 43.12; V2: 52.67, 42.96; V3: 40.13, 37.85) than 1 (V1: 15.44, V2: 15.59, V3: 39.74) or 3 (V1: 13.50, V2: 13.94, V3: 28.72) and only significant differences (p-value = 0.000) were observed for V1 and V2 when comparing scenarios 1 with 4 and 3 with 4, see Table 2.

### 3.2. Logistic Regression Model

The stepwise procedure was used to determine the logistic regression model capable of classifying the samples as normal or abnormal. Equation (1) is the governing equation of the model and the coefficients of each variable are listed in Table 3. All variables except V4 (*p*-value >0.05) are significant. As expected, V1 and V2 are the major contributors to the model.

y = cte1 × v1 + cte2 × v2 + cte3 × v3 + cte4 × v4 + cte5 × v5(1)

**Figure 3:**
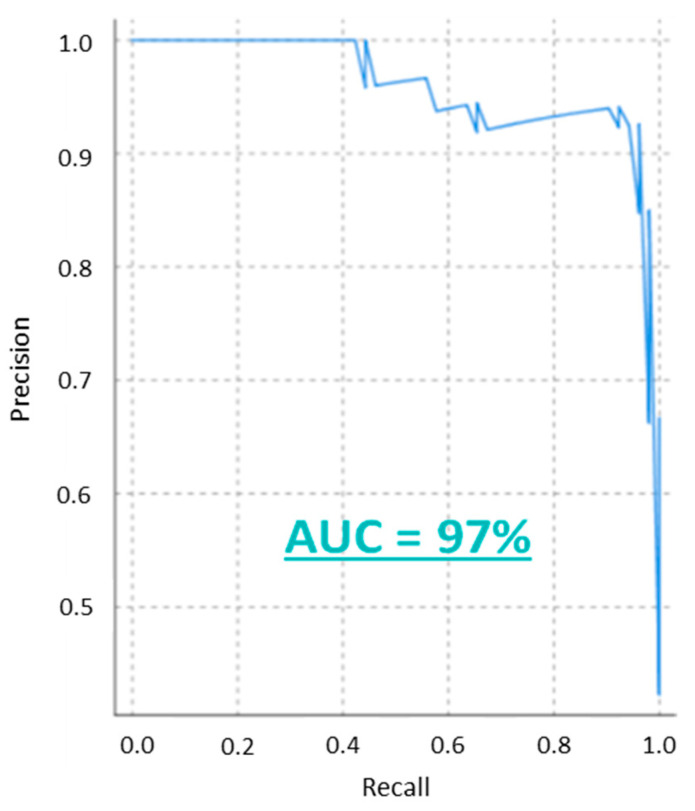
The precision-recall curve of the model with the calculated area under the curve.

To evaluate the performance of the model the confusion matrix was calculated (see Table 4) as well as several relevant performance indicators (see Table 5). Based on these results, the model can be considered to accurately distinguish between normal and abnormal values (93%), specificity (94%) and accuracy (94%) levels. Moreover, the model also has a high precision or PPV (92%), NPV (94%), recall (94%) and F-Score (92%). The AUC of the precision-recall curve is equal to 97%, see Figure 3. Additional parameters to evaluate the discriminative power of the model were also calculated. Parameters like the LR+ (16.38), the LR− (0.08), the DOR (201) and the YI (0.87) reveal the high discriminative power of the model.

Finally, it is worth noting that the processing time of this algorithm is less than one minute on a conventional laptop and therefore making it feasible to be implemented in smart wearable devices with similar processing capabilities can be embedded in wearable devices without a significant increase in manufacturing cost.

## 4. Discussion

The main objective of this study is to develop a detection algorithm oriented to electrochemical biosensors, for long-term real-world monitoring, which can accurately stratify the risk of a patient suffering myocardial injury or tissue necrosis and applicable to current clinical practice. This algorithm has shown a promising high performance according to a broad number of indicators and has a turnover time of less than one minute in a conventional laptop, making it feasible to be easily implementable in wearable devices for home-based care.

The gold standard method to diagnose ACS (stable and unstable angina, myocardial infarction) is based on the measurement of troponin levels in blood samples because this contractile protein has shown to have the highest diagnostic and prognostic capability in comparison with the other cardiac biomarkers identified over the past four decades of research [16,17]. According to recently published guidelines [18], patients with suspicion of ACS will be ruled-in of the ED if the troponin levels are at least “one value higher than the 99th percentile value” or by the contrary ruled-out after the first negative troponin measurement. In other words, the admission or discharge of a patient will be decided after comparing his troponin levels with a clinical decision cut-off value. Consequently, this parameter is key in current clinical practices. This is corroborated by the fact that the recommended reperfusion therapies are based on the type of ACS (e.g., mainly diagnosed based on troponin levels and electrocardiogram (ECG)) and on other complementary criteria (e.g., patient suffering contraindications, acute heart failure, hemodynamic instability or cardiogenic shock, persistent chest pain, life-threatening arrhythmias or cardiac arrest and time from symptoms) [16]. 

Current clinical cut-off values are established based on the sensitivity of the troponin assay and not according to gender, age, disease or comorbidities. The general reference values are approximately 0.03 μg/L and 0.06 ng/L for sensitive and high-sensitive assays, respectively. However, in the latter group, the sensitivity is so high that this value may vary among manufacturers due to the patient distribution and prevalence of the disease in the studied cohort. Regrettably, this has a greater impact on high-risk patients (e.g., diabetic patients, chronic kidney disease patients, elderly patients and heart failure patients) which demand a rapid and secure diagnosis and it might not be achievable due to abnormal baseline values or even because of asymptomatic conditions. Using personalized data acquire from minimally invasive wearable solutions can overcome these inconveniences and save health care expenditure by reducing the event to diagnosis time delay, crucial in this time-dependent life-threatening scenario, and the use of unnecessary resources [5]. 

Electrochemical sensors due to cheap manufacturing and high capacity for miniaturization can be ideal for the development of point of care devices or even wearable devices to monitor troponin levels in blood samples. Multiple efforts have been made in improving the biosensor component. One of those approaches is based on the development of different immobilization strategies or working principles. In Table 1 is summarized a list of them. However, this approach can be associated with high research and development costs and might not contribute to developing trustworthy wearable tools for home-based care. For example, a recently published systematic review with meta-analysis revealed that there is no conclusive evidence to suggest that high-sensitive troponin assay outperforms sensitive assays for free-risk ACS exclusion [25]. In contrast, limited efforts have been dedicated to the development of new detection algorithms which also play a relevant role as they are responsible to translate the changes in the measured signal(s) into troponin concentration. Even though some machine learning algorithms have been recently published [9,12], specially oriented to applications where multiple analytes must be identified for decision-making, the calibration method remains the most popular of them all. However, it has major drawbacks for its implementation in telemonitoring or wearable tools. 

To overcome or minimize these limitations the authors have developed a new detection algorithm that can increase the accuracy and safety of wearable solutions for long-term real-world monitoring and suitable for current clinical practice. The proposed algorithm is made up of two key components: feature extraction and variable normalization algorithm and the logistic regression model to classify samples as clinically normal or abnormal based on a clinical cut-off value. 

The feature extraction and data normalization algorithm are capable of identifying 60 features from the resulting current-potential curve generated from a cyclic voltammetry test. At this initial stage of development, it is recommended to extract numerous features not only to ensure the accuracy of the algorithm but especially to be accurate and robust enough when tested in real human samples. A robust and accurate algorithm will not only improve the biosensor but will transform a wearable device or a telemonitoring tool into a trustworthy home-based care solution. Some of the extracted features present significant differences between scenarios (i.e., sensor + PBS, sensor + coating, sensor + coating + blocking and sensor + coating + blocking + troponin). In particular, V1 and V2 are significantly greater in scenario 4 than in scenario 1 or 3, see Table 2. The other three variables (V3, V4 and V5) are also higher in scenario 4 than in any other one even though this difference is not statistically significant. This finding is relevant not only to contribute to the development of the logistic regression model but also to evaluate the state of the biosensor through timely scans. 

The resulting logistic regression model is the key element of this detection algorithm and was determined after applying the stepwise method in SPSS. The reference value for sensitive assays was used as the current clinical decision threshold in this manuscript, 0.03 μg/L. The resulting equation, Equation (1), is made up of five variables or predictors. All except V4 (*p*-value > 0.05) and as expected V1 and V2 are the major contributors, see Table 3. The model has a high performance according to the confusion matrix and to a broad number of calculated performance indicators, see Table 4 and Table 5, respectively. The accuracy, specificity, sensitivity, precision, recall and F-score are all over 90%. The fact that precision, recall and F-score are similar reveals that the model is balanced. In other words, the number of false-negative and false-positive are in both cases low and similar as it can be appreciated from the confusion matrix. The slight difference in accuracy in favor of the normal samples could be explained by the fact that the model was developed using an unbalanced dataset. By doing so the authors mimic the reality of ACS diagnosis in ED. In the UK, 70% of the patients with chest pain are discharged from ED. This percentage increases to approximately 90% in the USA [3,26]. 

Additional indicators commonly used to evaluate diagnostic tests were also calculated [22,23,24]. The LR+ and LR−measure the influence of a result on the probability while the DOR estimates the discriminative ability of the test. In this case, the indicators yield 16.38, 0.082 and 201, respectively. According to Ana-Maria Simundic et al. [23], LR+ and LR−values above 10 and below 0.01 suggest a good discriminative model. This is corroborated with a YI (0.866) close to one and by the AUC (97%) of the precision-recall curve, see Figure 3. 

This algorithm can also be easily tailored, based on personalized data, by modifying the cut-off value to better adapt to the needs and circumstances of the patient. In this context, the performance of the model was evaluated for several cut-off values. According to sample distribution, no changes are expected for cut-off values ranging between 0.03–0.06 μg/L. However, if the value is increased to 0.1 μg/L the performance of the model decreases, see Table S3 and Figure S1 of the Supplementary Material. Despite an AUC of the precision-recall curve equal to 88% the accuracy calculated from the confusion matrix (92% and 42% for normal and abnormal samples, respectively) was not as desired as it should be for this life-threatening scenario. This unbalanced performance could contribute to increasing the number of visits to the ED by rising the number of false-positive. 

According to the aforementioned findings, the algorithm not only has shown good performance in classifying between normal and abnormal samples, over a broad range of cut-off values, but also the capability of differentiating the conditions of the biosensor between scenarios. While the former can contribute to tailoring each device based on personalized data (e.g., age, gender, disease and comorbidities), the latter can be used as a quality management test to ensure the correct state of the biosensor before performing the corresponding readings. Both features indeed contribute to the development of trustworthy wearable or telemonitoring tools for long-term and real-world monitoring which can be implemented in current clinical practice. Even though the sample size is relatively small, and that some performance is expected to be lost when tested with real human blood samples, the preliminary results suggest that it is worth to pursue its development and optimization.

## 5. Conclusions

The proposed algorithm has a performance greater than 90% in stratifying the risk of a patient from suffering myocardial damage/tissue necrosis by measuring biomarker levels in small-volume biological samples, it has also shown its ability to work as a quality control tool by detecting differences in the surface of the biosensor and is aligned with current clinical practices. These not only mitigate the limitations of the calibration curve procedure but more important of all contributes to the development of trustworthy wearable tools for long-term and real-world monitoring oriented to home-based care. More research is indeed needed to optimize its performance in real blood samples and in making it broadly applicable. This latter is an expensive and complex task because of the lack of standardization in the field of biomarker assays.

## Figures and Tables

**Table 1:** List of electrochemical solutions capable of measuring troponin levels. Adapted from [7,8].

Biomarker	Methodology	Assay Principle	Concentration Range	Limit of Detection (LOD)
cTnT	NH2-CNT-SPEs/polyethyleneterephtalate (PET)/NHS-EDC-anti-cTnT/glycine/cTnT	Amperometric	0.0025–0.5 ng/mL	0.0035 ng/ml
cTnT	Gold (Au)/polyethyleneimine (PEI)/carboxylated CNTs (COOH-CNT)/ANTI-cTnT/glycine/ cTnT		0.1–10 ng/mL	0.033 ng/mL
cTnT	GCE/o-aminobenzoic acid (poly-o-ABA)/EDC/NHS/anti-cTnT/ethanolamine/ cTnT		0.05–5 ng/mL	0.016 ng/mL
cTnT	SPE/polyethylene terephtalate (PTE)/anti-cTnT/biotin/glutaraldehyde (glu)/streptavidin microsphere/glycine/cTnT/HRP-conjugated anti-cTnT		0.1–10 ng/mL	0.2 ng/mL
cTnI	Interdigitatedarray (IDA) chip/polydimethylsiloxane (PDMS)/NHS/BSA anti-cTnI/protein/ cTnI/alkalinephosphatase (AP)-labeled anti-cTnI/enzyme substrate (PaPP)		0.2 ng/mL–10 µg/mL	148 pg/mL
cTnI	PDMS-GNP composite/anti-cTnI and anti-CRP (Ab1)/BSA/CdTe and ZnSe quantum dots-anti-cTnI and anti-CRP (Ab2)		0.01–50 µg/mL	5 amol
cTnI	Microfluidic channel/EDC and SNHS/Branched polyethylenimine (BPEI)/BPEI activation with GA/anti- cTnI/BSA/cTnI/biotinylated detection antibody/GOx-avidin		NA	25 pg/mL
cTnI	SPE/AuNPs/anti-cTnI/BSA/cTnI	Capacitance	0.2–12.5 ng/mL	0.2 ng/mL
cTnT	An electrode/self-assembled monolayer/glutaredehyde/anti-cTnT/glysin/cTnT		0.07–6.38 ng/mL	NA
cTnT	Increase of low-frequency capacitance between two Al electrodes after Ab-Ag interaction		0.01–5 ng/mL (PBS) 0.07–6.83 ng/mL (serum)	NA
cTnI	Indium tin oxide (ITO)/Gold nanoparticles (GNPs)/anti-cTnI/cTnI/NHRP-conjugated anti-cTnI	Open circuit potential	1–100 ng/mL	NA
cTnT	cTnT/carboxylated, MWCNT/acrylamide (AAM), N,N-methylenebisacrylamide (NNMBA, cross-linker) and ammonium persulphate (APS, initiator)/cTnT	Potentiometric	1.41–20.68 µg/mL	0.16 µg/mL
cTnI	Au electrode/PANI nanowire integrated with microfluidic channels/anti-cTnI/cTnI	Conductance	NA	250 fg/mL
cTnT	GCE/(E)-1-decyl-4-[(4-decyloxyphenyl)diazenyl] pyridinium bromide (Br-Py)film/gold nanoparticles (AuNP) stabilized in a water-soluble 3-n-propyl-4-picolinium silsesquioxane chloride (Si4Pic + Cl−)/anti cTnT/glycine/cTnT	Cyclic voltammetry and impedance	0.1–0.9 ng/mL	0.076 ng/mL
cTnI	Interdigitated electrode surface/graphene-ABA nano composite/anti-hcTnI/cTnI		0.1–1 ng/mL	0.01 ng/mL
cTnT	GCE/I-Py/CTS-AuNP/anti-cTnT/glycine/cTnT	Cyclic voltammetry	0.2–1 ng/mL	0.1 ng/mL
cTnI	Au electrode modified with a mixed SAM where biotinylated antibodies were linked through neutravidin	Impedance	10–13–10–7 mol/L	10–13 mol/L
cTnI	PANI electrodeposited on patterned screen-printed paper electrodes. PANI oxidation current change after an immunological reaction	Cyclic voltammetry	1–100 ng/mL	NA
cTnT	Amine-functionalized CNT-SPEs platforms	Differential Pulse Voltammetry	0.0023 ng/mL–0.5 ng/mL	0.0035 ng/mL

AuNP: gold nanoparticles, cTnT: cardiac troponin isoform T, cTnI: cardiac troponin isoform I, CNT: carbon nanotube, PDMS: polydimethylsiloxane, Ab: antibody, Ag: antigen, PANI: polyaniline, SPE: screen-printed electrode, BSA: bovine serum albumin, HRP: horseradish peroxidase, EDC: (1-ethyl-3-(3-dimethylamino propyl/carbodiimide), NHS: *N*-hydroxysuccinimide, SNHS: *N*-hydroxysulfosuccinimide, SAM: self-assembled monolayer, GCE: glassy carbon electrode, PAPP: p-Aminophenyl phosphate, amol: 10^-18^ moles, NA: not available.

**Table 2:** Mann-Whitney U test: differences in ranks between relevant scenarios.

Variables	Scenario 1		Scenario 4		Mann-Whitney	Scenario 3		Scenario 4		Mann-Whitney
	Ranks	Sum	Ranks	Sum	*p*-Value	Ranks	Sum	Ranks	Sum	*p*-Value
V1	15.44	417.00	52.75	2743.00	0.000	13.50	243.00	43.12	2242.00	0.000
V2	15.59	421.00	52.57	2739.00	0.000	13.94	251.00	42.96	2234.00	0.000
V3	39.74	1073.00	40.13	2087.00	0.942	28.72	517.00	37.85	1968.00	0.101
V4	44.44	1200.00	37.89	1960.00	0.215	40.94	737.00	33.62	1748.00	0.186
V5	46.74	1262.00	36.50	1898.00	0.060	39.83	717.00	34.00	1768.00	0.295

**Table 3:** Summary of the logistic regression model.

Variables		B	Standard Error	Sig.
	V1	−31.356	15.141	0.038
	V2	43.863	17.369	0.012
	V3	5.150	1.366	0.000
	V4	−3.361	3.008	0.264
	V5	−8.577	2.103	0.000
	Constant	2.429	1.098	0.027

**Table 4:** Confusion matrix of the proposed model.

	Prediction		Accuracy
Observations	Normal	Abnormal	%
Normal	67	4	94
Abnormal	4	48	92
Total			94

**Table 5:** Performance of the diagnostic model according to several key performance indicators.

Performance Indicators.	Value
Accuracy	94.00%
Specificity	94.37%
Recall = Sensitivity	92.31%
PPV	92.31%
NPV	92.31%
AUC	97.00%
F-Score	92.31%
YI	0.87
LR+	16.38
LR−	0.082
DOR	71.64%

PPV: positive predictive value, NPV: negative predictive value, AUC: area under the curve, YI: Youden Index, LR+: Likelihood ratio positive, LR−: Likelihood ratio negative, DOR: diagnostic odd ratio.

## Data Availability

The data presented in this study are available on request form the corresponding author. The data are not publicly available due to the fact that the algorithm might be suitable for IP protection.

## Supplementary Materials

The following are available online at https://www.mdpi.com/2306-5354/8/2/28/s1, Figure S1: Precision-recall curve and the calculated area under the curve (AUC) for a cut-off value of 0.1 µg/L, Table S1: Mann-Whitney test for scenario 1, 3 and 4, Table S2: Mann-Whitney test for clinically normal and abnormal samples, Table S3: Confusion matrix for the proposed algorithm but with a cut-off value of 0.1 µg/L.
