# Supplementary material for: Development of a New Detection Algorithm to Identify Acute Coronary Syndrome Using Electrochemical Biosensors for Real-World Long-Term Monitoring
Source: Bioengineering (Basel). 2021 Feb 20;8(2):28. doi: 10.3390/bioengineering8020028 (PMC7924205; doi:10.3390/bioengineering8020028)
Supplement: Supplementary file 1 [file bioengineering-08-00028-s001.pdf]

Supplementary Material: In the following pages are presented the results of the statistic analyses for the rest of the variables not included in the main text. Tables S1 and S2 summarize the results of the Mann-Whitney test perform to identify which variables present significant differences under the different scenarios considered. Lastly, Table S3 and Figure S1 correspond to the performance of the model when the cut-off values is equal to 0.1 mg/L.

Table S1. Mann-Whitney test for scenario 1, 3 and 4

	Scenario 1		Scenario 4		Mann-Whitney	Scenario 3		Scenario 4		Mann-Whitney
Variable	Rank	Sum Rank	Rank	Sum Rank	p-value	Rank	Sum Rank	Rank	Sum Rank	p-value
V6	15.85	428.0	52.54	2732.0	0.000	12.44	244.0	43.48	2261.0	0.000
V7	17.78	480.0	51.54	2680.0	0.000	16.11	290.0	42.21	2195.0	0.000
V8	55.02	1485.5	32.20	1674.5	0.000	47.44	854.0	31.37	1631.0	0.000
V9	16.63	449.0	52.13	2711.0	0.000	23.17	417.0	39.77	2068.0	0.000
V10	43.26	1168.0	38.31	1992.0	0.579	42.78	770.0	32.98	1715.0	0.210
V11	20.52	554.0	50.12	2606.0	0.000	17.53	315.5	41.72	2169.5	0.000
V12	34.02	918.5	43.11	2241.5	0.385	35.00	630.0	35.67	1855.0	0.412
V13	15.48	418.0	52.73	2742.0	0.000	14.06	253.0	42.92	2232.0	0.000
V14	37.98	1025.5	41.05	2134.5	0.033	43.72	787.0	32.65	1698.0	NA
V15	20.44	552.0	50.15	2608.0	0.000	33.06	595.0	36.35	1890.0	0.412
V16	37.52	1013.0	41.29	2147.0	0.180	20.08	361.5	40.84	2123.5	0.000
V17	15.44	417.0	52.75	2743.0	0.000	14.06	253.0	42.92	2232.0	0.000
V18	15.48	418.0	52.73	2742.0	0.000	14.06	253.0	42.92	2232.0	0.000
V19	26.13	705.5	47.20	2454.5	0.022	44.44	800.0	32.40	1685.0	NA
V20	35.28	952.5	42.45	2207.5	0.022	43.11	776.0	32.87	1709.0	NA
V21	23.33	630.0	48.65	2530.0	0.001	40.17	723.0	33.88	1762.0	0.412
V22	38.41	1037.0	40.83	2123.0	0.303	28.78	518.0	37.83	1967.0	0.056
V23	39.20	1058.5	40.41	2101.5	0.048	15.36	276.5	42.47	2208.5	0.000
V24	37.04	1000.0	41.54	2160.0	0.694	24.83	447.0	39.19	2038.0	0.014
V25	27.37	739.0	46.56	2421.0	0.022	43.33	780.0	32.79	1705.0	0.056
V26	22.33	603.0	49.17	2557.0	0.006	31.28	563.0	36.96	1922.0	0.172
V27	25.59	691.0	47.48	2469.0	0.001	15.61	281.0	42.38	2204.0	0.000
V28	15.41	416.0	52.77	2744.0	0.000	14.39	259.0	42.81	2226.0	0.000
V29	15.44	417.0	52.75	2743.0	0.000	13.50	243.0	43.12	2242.0	0.000
V30	15.37	415.0	52.79	2745.0	0.000	14.33	258.0	42.83	2227.0	0.000
V31	15.26	412.0	52.85	2748.0	0.000	10.50	189.0	44.15	2296.0	0.000
V32	15.26	412.0	52.85	2748.0	0.000	10.56	190.0	44.13	2295.0	0.000

V33	15.26	412.0	52.85	2748.0	0.000	10.56	190.0	44.13	2295.0	0.000
V34	15.67	423.0	52.63	2737.0	0.000	14.72	265.0	42.69	2220.0	0.000
V35	64.33	1737.0	27.37	1423.0	0.000	56.28	1013.0	28.31	1472.0	0.000
V36	15.44	417.0	52.75	2743.0	0.000	11.50	207.0	43.81	2278.0	0.000
V37	15.44	417.0	52.75	2743.0	0.000	11.50	207.0	43.81	2278.0	0.000
V38	16.22	438.0	52.35	2722.0	0.000	14.39	259.0	42.81	2226.0	0.000
V39	63.78	1722.0	27.65	1438.0	0.000	56.61	1019.0	28.19	1466.0	0.000
V40	64.19	1733.0	27.44	1427.0	0.000	55.56	1000.0	28.56	1485.0	0.000
V41	15.59	421.0	52.67	2739.0	0.000	10.50	189.0	44.15	2296.0	0.000
V42	17.44	471.0	51.71	2689.0	0.000	32.89	592.0	36.40	1893.0	0.172
V43	44.44	1200.0	37.69	1960.0	0.032	40.94	737.0	33.62	1748.0	0.056
V44	17.44	471.0	51.71	2689.0	0.000	32.89	592.0	36.40	1893.0	0.172
V45	15.52	419.0	52.71	2741.0	0.000	13.94	251.0	42.96	2234.0	0.000
V46	16.93	457.0	51.98	2703.0	0.000	15.11	272.0	42.56	2213.0	0.000
V47	15.52	419.0	52.71	2741.0	0.000	13.94	251.0	42.96	2234.0	0.000
V48	16.93	457.0	51.98	2703.0	0.000	15.11	272.0	42.56	2213.0	0.000
V49	59.81	1615.0	29.71	1545.0	0.000	56.56	1018.0	28.21	1467.0	0.000
V50	63.67	1719.0	27.71	1441.0	0.000	55.56	1000.0	28.56	1485.0	0.000
V51	62.89	1698.0	28.12	1462.0	0.000	59.72	1075.0	27.12	1410.0	0.000
V52	16.26	439.0	52.33	2721.0	0.000	11.56	208.0	43.79	2277.0	0.000
V53	15.41	416.0	52.77	2744.0	0.000	11.28	203.0	43.88	2282.0	0.000
V54	15.44	417.0	52.75	2743.0	0.000	11.56	208.0	43.79	2277.0	0.000

NA: not available

Table S2. Mann-Whitney test for clinically normal and abnormal samples

Variables	Normal (0) / Abnormal (1)	N	Rank	Sum Rank	Mann-Whitney p-value
V1	0	78	46.61	3635.5	0.000
	1	45	88.68	3990.5	
V2	0	78	46.29	3610.5	0.000
	1	45	89.23	4015.5	
V3	0	78	58.92	4595.5	0.366
	1	45	67.34	3030.5	
V4	0	78	62.62	4884.5	0.186
	1	45	60.92	2741.5	
V5	0	78	65.97	5145.5	0.050
	1	45	55.12	2480.5	
V6	0	78	46.07	3593.5	0.000
	1	45	89.61	4032.5	
V7	0	78	47.37	3694.5	0.000
	1	45	87.37	3931.5	
V8	0	78	72.53	5657.5	0.000
	1	45	43.74	1968.5	
V9	0	78	51.85	4044.5	0.000
	1	45	79.59	3581.5	
V10	0	78	64.63	5041.5	0.052
	1	45	57.43	2584.5	
V11	0	78	51.88	4047.0	0.000
	1	45	79.53	3579.0	
V12	0	78	56.48	4405.5	0.028
	1	45	71.57	3220.5	
V13	0	78	46.98	3664.5	0.000
	1	45	88.03	3961.5	
V14	0	78	65.05	5074.0	0.281
	1	45	56.71	2552.0	
V15	0	78	56.43	4401.5	0.000
	1	45	71.66	3224.5	
V16	0	78	56.23	4386.0	0.009
	1	45	72.00	3240.0	
V17	0	78	46.92	3659.5	0.000
	1	45	88.14	3966.5	
V18	0	78	46.80	3650.5	0.000
	1	45	88.34	3975.5	
V19	0	78	60.96	4754.5	0.458
	1	45	63.81	2871.5	
V20	0	78	63.97	4990.0	0.719
	1	45	58.58	2636.0	
V21	0	78	59.83	4667.0	0.063
	1	45	65.76	2959.0	
V22	0	78	59.27	4623.0	0.095
	1	45	66.73	3003.0	
V23	0	78	52.85	4122.0	0.000
	1	45	77.87	3504.0	
V24	0	78	58.20	4539.5	0.034
	1	45	68.59	3086.5	

V25	0	78	62.13	4846.5	0.994
	1	45	61.77	2779.5	
V26	0	78	55.20	4305.5	0.000
	1	45	73.79	3320.5	
V27	0	78	49.84	3887.5	0.000
	1	45	83.08	3738.5	
V28	0	78	47.12	3675.5	0.000
	1	45	87.79	3950.5	
V29	0	78	46.26	3608.5	0.000
	1	45	89.28	4017.5	
V30	0	78	47.07	3671.5	0.000
	1	45	87.88	3954.5	
V31	0	78	45.47	3546.5	0.000
	1	45	90.66	4079.5	
V32	0	78	45.76	3569.5	0.000
	1	45	90.14	4056.5	
V33	0	78	45.76	3569.5	0.000
	1	45	90.14	4056.5	
V34	0	78	46.56	3631.5	0.000
	1	45	88.77	3994.5	
V35	0	78	77.44	6040.5	0.000
	1	45	35.23	1585.5	
V36	0	78	46.52	3628.5	0.000
	1	45	88.83	3997.5	
V37	0	78	46.52	3628.5	0.000
	1	45	88.83	3997.5	
V38	0	78	46.58	3633.5	0.000
	1	45	88.72	3992.5	
V39	0	78	77.42	6038.5	0.000
	1	45	35.28	1587.5	
V40	0	78	77.17	6019.5	0.000
	1	45	35.70	1606.5	
V41	0	78	46.40	3619.5	0.000
	1	45	89.03	4006.5	
V42	0	78	53.01	4134.5	0.000
	1	45	77.59	3491.5	
V43	0	78	62.62	4884.5	0.186
	1	45	60.92	2741.5	
V44	0	78	53.01	4134.5	0.000
	1	45	77.59	3491.5	
V45	0	78	46.52	3628.5	0.000
	1	45	88.83	3997.5	
V46	0	78	48.17	3757.5	0.000
	1	45	85.97	3868.5	
V47	0	78	46.52	3628.5	0.000
	1	45	88.83	3997.5	
V48	0	78	48.17	3757.5	0.000
	1	45	85.97	3868.5	
V49	0	78	75.37	5878.5	0.000
	1	45	38.83	1747.5	
V50	0	78	76.42	5960.5	0.000

	1	45	37.01	1665.5	
V51	0	78	78.54	6126.5	0.000
	1	45	33.32	1499.5	
V52	0	78	46.28	3609.5	0.000
	1	45	89.26	4016.5	
V53	0	78	46.06	3592.5	0.000
	1	45	89.63	4033.5	
V54	0	78	46.66	3639.5	0.000
	1	45	88.59	3986.5	

Different cut-off values were studied to determine their impact on the performance of the model. The upper limit has been found to be 0.1 mg/L. Despite the area under the curve (AUC) of the precision-recall plot is equal to 88%, the confusion matrix clearly is unbalanced towards classifying the clinically normal samples. This is not the most desirable performance for this life-threatening scenario. Excessive number of false positive can be reported in this case. Therefore, it is recommended to modify the model to one more appropriate. This will be studied in future research when the number of samples will be increased and cross-validated with real-blood samples.

Table S3. Confusion matrix for the proposed algorithm but with a cut-off value of 0.1 µg/L.

Observations	Prediction		Accuracy %
	**Normal**	**Abnormal**	
Normal	84	5	94
Abnormal	20	15	43
Total			81

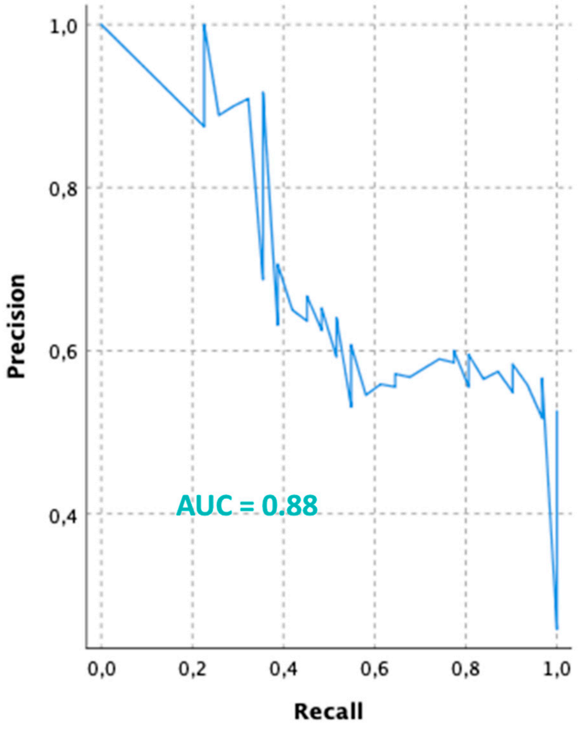

Figure 1. Precision-recall curve and the calculated area under the curve (AUC) for a cut-off value of 0.1 µg/L.
